# Uncovering new lineages in the Sunda pangolin (*Manis javanica*) with museum mitogenomics

**DOI:** 10.1098/rsbl.2025.0222

**Published:** 2025-09-10

**Authors:** Arlo Hinckley, Mary Faith C. Flores, Nurul Inayah, Melissa T. R. Hawkins

**Affiliations:** ^1^ Department of Vertebrate Zoology, Division of Mammals, Smithsonian National Museum of Natural History, Washington, DC, USA; ^2^ Departamento de Zoología, Universidad de Sevilla, Seville, Spain; ^3^ Museum Zoologicum Bogoriense, Research Center for Biosystematics and Evolution, National Research and Innovation Agency, Cibinong, Indonesia

**Keywords:** Mammalia, Mentawai, museomics, Pholidota, phylogeography, southeast Asia, systematics, wildlife conservation, conservation genetics

## Abstract

Accurately identifying evolutionarily significant units (ESUs) is crucial for conservation planning, especially for species like pangolins threatened by overhunting and habitat loss. ESUs help categorize different pangolin populations, aiding in understanding their genetic diversity and distribution, which is vital for targeted conservation efforts. This research generated mitochondrial genomes from historical museum specimens of Sunda pangolins (*Manis javanica*) from underrepresented locations, uncovering a new evolutionary lineage from the Mentawai Islands that diverged from Indochina and west Sundaland populations around 760 000 years ago. This population thereby represents a divergent ESU with a small distribution, important for conservation planning. The novel sequences provide resources for forensic labs tracing the origin of confiscated scales and shed light into the potential distribution of the ‘mysterious pangolin’. Additionally, this research confirmed the presence of the two major *M. javanica* lineages in Java and extended the known distribution of the eastern clade to Bali and East Kalimantan. Our findings potentially suggest a recent bottleneck and postglacial expansion of pangolins across Indochina and west Sundaland. Further investigation with genomic and morphological evidence, contact area sampling and type sequencing will be required to evaluate the taxonomic status of different *M. javanica* lineages and *M. culionensis*.

## 1. Introduction

Accurately identifying evolutionarily significant units (ESUs) is essential to informing conservation planning, particularly for species threatened by ongoing overhunting and habitat loss such as is the case of pangolins [1,2]. ESUs help in accurately identifying and categorizing different pangolin populations, which is essential for understanding their genetic diversity and distribution. This information is vital for developing effective conservation strategies, as it allows for targeted efforts to protect specific populations that may be at greater risk. Additionally, well-defined ESUs can aid forensic agencies in tracking and combating illegal wildlife trade, ensuring that conservation measures are both scientifically informed and practically effective [2].

Pangolins have regrettably become symbols of the illegal wildlife trade and are now considered the most trafficked mammals in the world [3]. Three of the five currently recognized Asian pangolin species (*Manis culionensis*, *M. javanica* and *M. pentadactyla*) are categorized as Critically Endangered, while a fourth species is Endangered (*M. crassicaudata*) [4–7]. The recently revalidated *Manis aurita* has not yet been formally assessed, but it is likely also Critically Endangered, given that it was split from *M. pentadactyla*, which already held that status prior to its range being divided [8–10]. A sixth species has been identified from trafficked scales from the illegal wildlife trade but not formally described due to the unknown provenance and the lack of a complete specimen [11,12]. It is both timely and important to discern the geographic region from where this species originates by sequencing vouchered specimens from unsampled locations. The resulting sequences will also provide georeferenced DNA sequences to inform conservation agencies and/or improve geographic provenance resolution of confiscated scales for forensic applications. Currently, the majority of nucleotide sequences in public databases like GenBank are not linked to voucher specimens with known geographic origin, which can result in taxonomic misinterpretations that have impacted genomic studies [2,13]. Furthermore, mtDNA data are spread across different repositories and frequently not available on GenBank [14,15], or not available in an accessible assembled sequence format, but as raw reads [2]. These confounding variables (challenging access to published data, lack of geographical information and the difficulty of identifying sequences of known and unknown provenance, e.g. trafficked scales) can limit forensic applications and conservation efforts.

The primary goal of this research is to generate mitochondrial genomes of historical museum specimens from locations previously unrepresented or poorly represented in public sequence repositories. This effort focuses on increasing sequences of the Sunda pangolin, *Manis javanica,* to inform conservation planning and bolster available data for wildlife forensics. By publishing these data for the international community, we are democratizing access to North American collection resources. Second, we aim to assess whether any cryptic lineages or potentially overlooked ESUs exist in currently unsampled geographic areas, particularly those associated with high levels of endemism—such as West Java, Bali and the Mentawai Islands (Indonesia), as well as the Annamite (spanning Laos and Vietnam) and the Cardamom ecoregions (shared by Cambodia and Thailand). A final goal is to investigate the presence of the ‘mysterious pangolin’ [11,12] across *Manis* spp. specimens from North American collections, to better understand its distribution and formally describe this elusive species, if detected.

## 2. Material and methods

**Figure 1.**
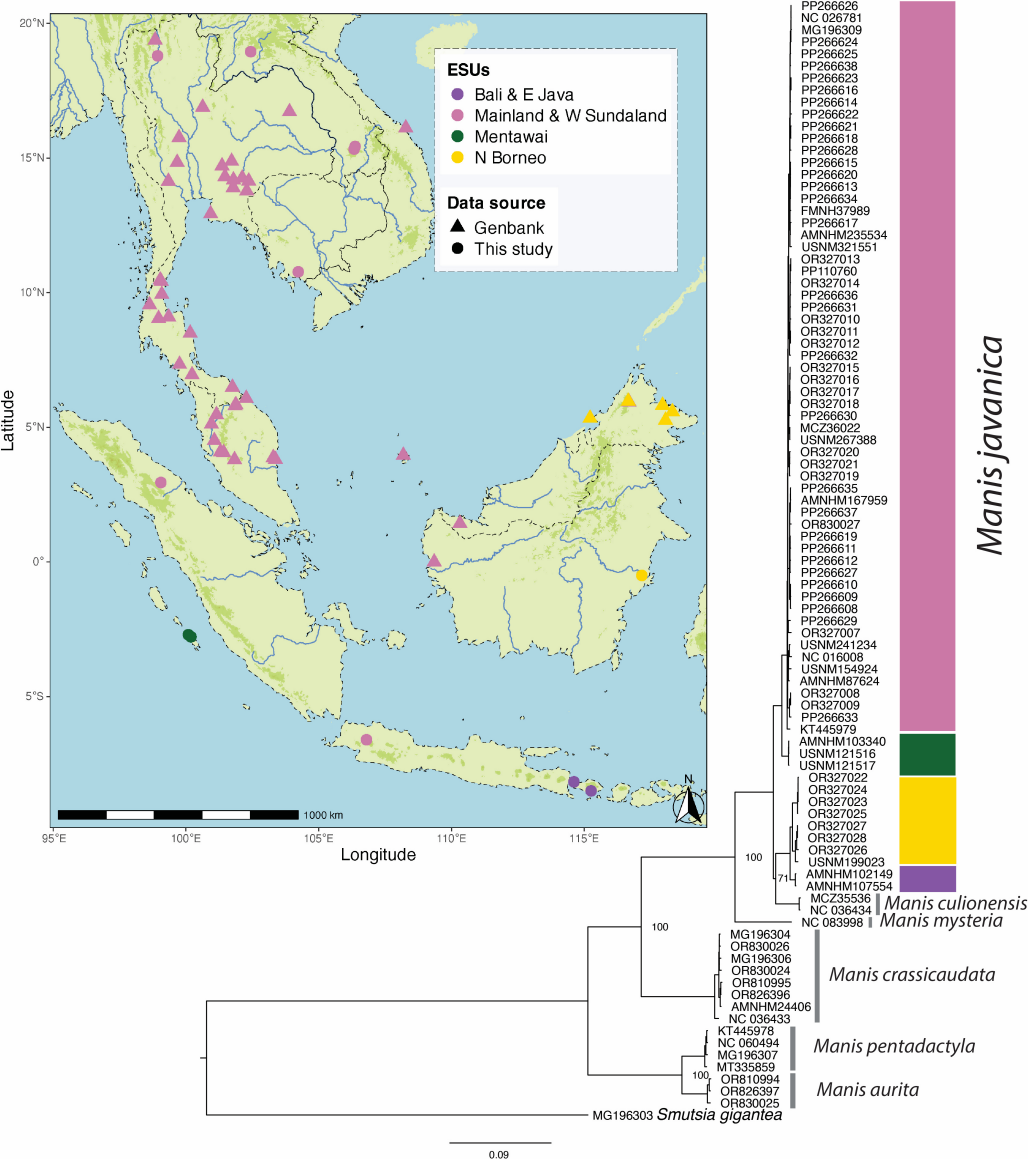
Map of southeast Asia displaying sampling localities with mitochondrial genome data (15 254 bp) for *Manis javanica*. Points represent new sequences generated in this study while triangles represent GenBank sequences. Different lineages are shown in distinct colours. Darker green shades indicate elevations higher than 800 m a.s.l. A maximum-likelihood consensus phylogeny of *Manis javanica* and *Manis culionensis* based on mtDNA genomes and reconstructed using IQ-TREE 2 is shown to the right of the map. Nodes with support values below 95% are represented by a square. The geographic origin of the study species is indicated by colours on the adjacent vertical bar, corresponding to the colours used in the map. Tip labels represent museum catalogue numbers or GenBank accession numbers (see electronic supplementary material, table S1). The species *M. javanica* exhibits two major clades, each further divided into two subclades. The ‘West’ clade comprises a subclade from mainland Asia, Sumatra, Natuna Islands and west/south Borneo (pink coloured points), and another subclade from Mentawai (green coloured points). The ‘East’ clade includes a subclade from north-east Borneo (yellow coloured points) and another from East Java and Bali (purple coloured points).

In total, 20 *Manis* spp. vouchered specimens—16 *M. javanica*, three *M. crassicaudata* and one *M. culionensis*—from novel unsequenced locations from regions with high levels of endemicity were sampled from North American museum collections under sterile conditions, following approvals for destructive sampling (figure 1; electronic supplementary material, table S1). These samples are from the following US museum collections: Smithsonian National Museum of Natural History (USNM; eight samples), American Museum of Natural History (AMNH; eight samples), Harvard Museum of Comparative Zoology (MCZ; two samples) and Field Museum of Natural History (FMNH; one sample; expanded in electronic supplementary material, table S1). Archival notes were examined for all museum specimens in order to document if these were collected in the wild or obtained in local markets, which was not an absolute determination if specimens were wild or local market origin, but to the best of our ability with available notes (electronic supplementary material, table S1). Skull osteocrusts, nasal turbinates, scales, claws, bone fragments, skin clips and liver or other soft tissues were sampled from the museum specimens and used in DNA extractions. All pre-PCR steps were conducted in the NMNH Historic DNA facility at the Museum Support Center. Skin, osteocrusts and other soft tissues were extracted using the Qiagen QIAamp DNA Mini kit protocol. Harder substrates such as scales and claws were extracted using a vacuum-manifold phenol–chloroform protocol. Single-stranded libraries were then constructed using a modification of the ‘single strand’ SRSLY Pico Plus Kit (ClaretBio) protocol described in [16] with half-volume reactions to reduce costs. Following library preparation, the libraries were quantified using a Qubit High Sensitivity Kit and pooled in equimolar ratios for shotgun sequencing. We targeted 10× coverage whole-genome sequencing, which provided a cost-effective approach for recovering complete mitochondrial genomes. For this purpose, we utilized a shared NovaSeq X system with 150 bp paired-end sequencing via the Oklahoma Medical Research Foundation NGS Core.

Adaptor removal and quality trimming were performed with Trimmomatic [17] with the sliding window parameter set to 5:20, read minimum length parameter to 30 bp, and leading and trailing to 5 for the remaining samples. Quality-trimmed reads were mapped to the closest available reference of each species with BWA-MEM (Li 2013): OR327028 (*M. javanica*) and OR826396 (*M. crassicaudata*). The output BAM files were sorted, PCR duplicates were removed with SAMtools [18] and libraries from the same specimens were merged. BAM files were imported to GENEIOUS where consensus sequences were called with minimum 3× coverage and 75% threshold.

Trailing N’s were removed, and two partially assembled mitogenome sequences with more than 50% of ambiguities were not considered for the core multiple sequence alignment. Consensus sequences were annotated in GENEIOUS using as a reference OR327028. Consensus sequences, along with all GenBank data for *M. javanica*, representatives of all other *Manis* species, and *Smutsia gigantea* (electronic supplementary material, table S1), for outgroup purposes, were aligned using the MAFFT v.7.450 GENEIOUS plug in [19] under default parameters. The control region was removed from the mitogenome assemblies due to poor assembly and low phylogenetic resolution [20]. Protein-coding regions were translated and inspected for frameshift mutations and for the presence of unexpected stop codons.

Maximum likelihood (ML) phylogenetic inference was conducted with IQ-TREE 2.3.1 [21]. Substitution model selection and the best-partition scheme were carried out with IQ-TREE’s ModelFinder implementation [22]. The input 53 partitions were merged into 10 partitions (see electronic supplementary material, Appendix), FreeRate heterogeneity model was considered, a relaxed clustering of 10% was selected, and each partition was allowed to have its own evolutionary rate (-m MFP+MERGE; electronic supplementary material, Appendix). ML tree reconstruction was run following the best-partition model. Edge-linked with proportional branch lengths (-spp) and unlinked-edge (-sp) partition models were run, with the former being selected due to its higher BIC scores (electronic supplementary material, Appendix). Ultrafast bootstrap support (UFBoot) was computed with 1000 bootstrap replicates. A second *cytochrome b* analysis was run following the same procedure but with three input partitions, one per each codon position. This analysis was run with the aim to put in context the sequences generated in this study with additional sequences from GenBank. After a first attempt, which yielded poor nodal support due to the inclusion of very short partial genbank sequences, a second analysis was run with complete *Cytb* sequences (1040 bp). Uncorrected pairwise genetic distances were computed with ‘ape’ [23] in R based on all available *cytochrome b* sequences in this gene dataset with a known geographic provenance. Additionally, a distance matrix table was computed with the IQTREE mitochondrial genome alignment on Geneious.

Divergence times were estimated with the mitochondrial genome dataset in BEAST2 v. 2.6.7 [24]. The mitochondrial genome alignment was split with AMAS [25] into the best partition schemes as identified by ModelFinder [22]. We conducted saturation plots with ‘ape’ in R [23] to assess the extent of substitution saturation in sequence data, and six partitions showing no signs of saturation with a total length of 11 401 bp were kept for downstream analyses (expanded in electronic supplementary material, Appendix S1). We unlinked site models and linked clock and tree models. We performed site model selection using a Bayesian approach with bModelTest, choosing between transition/transversion split and time-reversible models based on ModelFinder results, which varied across partitions, and applying empirical frequency priors [26]. We conducted exploratory analyses using both strict and lognormal (uncorrelated) relaxed molecular clock models. The coefficient of variation for the relaxed clock was approximately 0.3, indicating moderate rate heterogeneity among lineages and supporting the use of the relaxed lognormal clock model in subsequent analyses. We used the same two secondary calibrations and parameters described and applied in [1], which were derived from a well-resolved multilocus phylogeny [3]. Three independent runs of Markov chains (MCMC) for Monte Carlo simulations were run for 100 000 000 generations, with parameters and trees sampled every 5000 generations. Convergence was checked using TRACER 1.7.1 [27]. For each run, the first 20% of sampled trees were discarded as burn-in. These analyses trees were later merged with LogCombiner, and a maximum credibility consensus tree was generated with Treeannotator using median node heights.

## 3. Results

We successfully assembled complete mitochondrial genomes for 16 of the 19 samples’ (electronic supplementary material, table S1). Three specimens had previously failed in [14] (USNM 121516, 121517, 154924), highlighting the potential impact of improvements and protocols for museum genomics methods (e.g. SRSLY library prep) and increased sequencing coverage. Average coverage of mitogenomes ranged from 2× to 250×, with 6× as the threshold of being considered a ‘complete’ sequence. The mitochondrial genome alignment had 93 taxa—including 74 ingroup and 19 outgroup sequences—and 15 254 bp (IQTREE) or 11 510 bp (BEAST). The *cytochrome b* alignment had 295 taxa—including three ingroup and 292 outgroup sequences—and 1040 bp.

The interspecific relationships observed were consistent with those reported in previous studies [1,12,28]. The species *M. javanica* exhibited two major clades; each further divided into two subclades (figure 1; electronic supplementary material, figure S1). The ‘West’ clade comprised a subclade from mainland Asia, Sumatra, Natuna Islands and west/south Borneo (pink coloured points in figure 1), and another subclade from Mentawai (green coloured points in figure 1). The ‘East’ clade included a subclade from north-east Borneo (Sabah and East Kalimantan; yellow-coloured points in figure 1) and another from East Java and Bali (purple-coloured points in figure 1). The East clade of *M. javanica* was found to be sister to *M. culionensis*, although this relationship was weakly supported (Ultrabootstrap support: 71; posterior probabilities: 0.8). The extremely short internal branch at this node suggests a polytomy. *Cytochrome b (Cyt b*) and mitogenome (mtg) uncorrected genetic distances among the two major *M. javanica* clades (*Cyt b:* 2.4–4.2%; mtg: *ca* 3%; electronic supplementary material, figure S2) are similar to those between *M. culionensis* and *M. javanica* (*Cyt b:* 2.5–3.2%; mtg: *ca* 2.9%; electronic supplementary material, figure S2). Differentiation between the Mentawai and mainland Asia + west Sundaland lineages is lower (*Cyt b:* 1.5−2%; mtg: *ca* 1.3%; electronic supplementary material, figure S2).

The ‘West’ and ‘East’ clades of *M. javanica* diverged almost at the same time (*ca* 1.5 mya) as *M. javanica* and *M. culionensis* (*ca* 1.8 mya). The Mentawai Island population diverged from Indochina and west Sundaland populations around 760 000 years ago. The recently split *M. aurita* and its sister species *M. pentadactyla* diverged during the Early Pleistocene (*ca* 2.2 mya).

## 4. Discussion

By sequencing mitochondrial genomes of historical museum specimens from previously unrepresented locations in published data [1,3,9,14,15,29,30], this research uncovered a new evolutionary lineage of the Sunda pangolin (*M. javanica*) from the Mentawai Islands. The novel sequences provide new resources and increased geographical resolution for forensic labs tracing the origin of confiscated scales. Additionally, this work sheds some light into the potential distribution of the ‘mysterious pangolin’.

The Mentawai Archipelago represents a divergent evolutionary significant unit with a small distribution, which should be considered in conservation planning. The two Sunda Pangolin West and East lineages, referred as ‘MJA’/ ‘mainland’ and ‘MJB’/‘island’ in [28] have been estimated to diverged *ca* 300 000 years ago (Kya) according to population genomic based demographic model simulations without fossil calibrations [28], or estimated at approximately 1.56−1.81 mya ([1]; this study) based on mitogenome phylogenetics calibrated using external node ages derived from [3], which conducted multilocus-based phylogenetic inference with multiple fossil calibrations. The Mentawai lineage is part of the West/MJA clade, and it diverged from the other populations of this clade—mainland Asia, Sumatra and west Java—around 0.76 mya. This relatively recent divergence, together with a 1.5−2% uncorrected *Cytochrome b* distance from other populations within the clade, likely reflects intraspecific differentiation [31]. Such potential conspecificity is worth highlighting since many mammals found in the Mentawai are endemic to this archipelago and exhibit ancient divergences from their sister species [32–37]. Although geographically close to Sumatra, the Mentawai Islands were not directly connected by land bridges during periods of lower sea levels, due to the presence of a deep-sea trench that separates them [38,39]. This could suggest an overwater dispersal event between Mentawai and Sumatra at approximately 0.76 mya. Alternatively, since this dispersal event occurred during the Mid-Pleistocene Transition—a period marked by the formation of extensive ice sheets and pronounced sea-level lowstands—a land bridge via the Batu Islands offers a plausible route for dispersal [35,38,40,41]. The Batu Islands were connected to the exposed Sunda Shelf and Mentawai during periods when sea level dropped 75  m below present [40].

The close relationship between East Java—Bali and Sabah—East Kalimantan populations is surprising but not new, as [1] previously documented it for East Java and Sabah. This study extends this distribution to Bali and East Kalimantan. Remarkably, the East Java–Bali and East Borneo (East lineage) close relationship shows some degree of spatial congruence with that of white-rumped shama (*Copsychus malabaricus* complex) [42]. Separately evolving lineages white-rumped shama from East Java and East Borneo were shown to exhibit secondary gene flow suggesting some degree of physical connectivity among these regions, perhaps across exposed land bridges among landmasses during glacial cycles [42]. It is likely that *M. javanica* also dispersed between these two regions through exposed land bridges around 640 kya, during Middle Pleistocene sea-level low stands.

This study also confirms for the first time the presence of the two major *M. javanica* lineages in Java. This finding might explain why Javan samples of unknown provenance in [30] were not monophyletic in their SNP phylogeny. Testing admixture levels between these major lineages across east–west Java and Borneo with genomic evidence would be of interest to assess their taxonomic status. This has not been adequately assessed yet with nuclear evidence, as the nuclear SNP analyses in [1,30] were based on small sample sizes for some populations and relied primarily on seized samples, with wild-origin samples limited to east Java (east clade) and west and south Borneo (west clade). If we look at the evolutionary history of other Sundaic lowland forest mammals, different scenarios have taken place when it comes to patterns of divergence and mitonuclear discordance in these landmasses. In Java, Oriental shrews (*Crocidura orientalis*) show mitochondrial but little nuclear differentiation across this landmass, while Bartels’s spiny rats (*Crunomys bartelsii*) exhibit both mitochondrial and nuclear differentiation between west and central Java [43,44]. In Borneo, shrews (*C. foetida sensu lato*) exhibit both mitochondrial and nuclear differentiation between the northeast and remaining island, that is spatially congruent with *M. javanica* [43]. In fact, east–west differentiation across Borneo has emerged as a widespread biogeographic pattern over the past decade, with consistent evidence across diverse and distantly related taxa—including colugos, lesser mouse deer, Sunda rats and squirrels [14,35,45]. In sharp contrast to Java and Borneo, the remarkably low genetic structure across mainland Asia and Sumatra is inconsistent with the evolutionary histories seen in many mammals [14,43,46–48]. Such little structure across such a large and geographically complex region potentially suggests a recent bottleneck and postglacial expansion of pangolins across Indochina and west Sundaland, or alternatively, high female-mediated gene flow across this region. The latter seems less likely given that pangolins are solitary and territorial, so male-biased dispersal would be expected by default—not female-mediated gene flow. Finally, this study also highlights for the first time very little structure across central and northern populations of *M. culionensis*.

This research suggests that if the ‘mysterious pangolin’ occurs in Sundaland or southern Indochina, its range may be restricted, and it is likely highly endangered, as the present, and other studies [1,3,9,14,15,29] have surveyed much of the known distribution of *Manis* spp. The Leuser ecosystem in north Sumatra, or the Dalat and Dak Lak plateaus in south Viet Nam are both areas of high endemicity [48], where *Manis* spp. has not been sampled—[14] incorrectly placed their sequenced specimen in southern Viet Nam when it is actually from central Viet Nam. Similarly, most of Myanmar has not been sampled due to past and ongoing political instability. Alternatively, the mysterious pangolin might be sympatrically distributed with other congeneric species but in much lower population density or in a less accessible habitat (e.g. higher degree of scansoriality).

Divergence exhibited among east and west *M. javanica* clades (*ca* 1.55 mya) seems as nearly as much as that with *M. culionensis* (*ca* 1.75 mya) in this study and [1,3]. *Cytochrome b* uncorrected genetic distances among these two major clades are between 2.4% and 4.2%, while those of *M. culionensis* and *M. javanica* are between 2.5% and 3.2% (electronic supplementary material, figure S2). If this pattern is confirmed with genomic and morphological evidence, potential solutions would be to lump both species and consider *M. culionensis* de Elera, 1915 a junior synonym of *M. javanica* Desmarest, 1822—which seems unlikely since these lineages are morphologically distinct [49] and diverged between 1.75 (this study and [1,3]) and 3 mya [12]—or to split *M. javanica* east and west clades. The splitting will require type sequencing since the type locality of *M. javanica* is ‘Java’, an island where both major lineages are present. Similarly, the oldest alternative name available for the other potential species would be *Manis leptura* (Blyth 1842), a name that lacks a type locality, so linking it to a major lineage would also require type sequencing, if the type—which is not specified in the original description—can be located. If nuclear evidence supports the currently recognized species limits, it might be worth treating as subspecies the east and west *M. javanica* lineages as proposed in [1], provided these can be assigned to the previously mentioned available names. Governments, forensic labs and conservation agencies could manage these incompletely separately evolving lineages more properly if they had a trinomial name [50].

Identifying illegally traded species and tracing their origins are crucial steps in understanding and combating illegal wildlife trade [2]. The vouchered specimens from novel unsequenced locations (Bali and Mentawai (Indonesia), Pakistan, Lao P.D.R., Cambodia) and associated mitogenome sequences, will be highly informative for forensics, captive breeding and reintroduction programmes. In this study, seized scale sequences from North Sumatra (MG825513, MG825505) and East Java (MG825497, MG825528) clustered with sequences from those regions (USNM267388 and MCZ36022 and AMNHM102149, respectively), suggesting local poaching by traffickers, while scales confiscated in Hong Kong (OR256862, OR256997-8, OR257024) clustered with samples from East Kalimantan, Borneo (USNM199023) revealing an illegal trade route among these regions. Surprisingly, not a single seized scale sample available on GenBank is from the Mentawai (electronic supplementary material, figure S1). This could indicate that populations in this archipelago may be at very low densities or locally extinct, that illegal traffickers are not targeting this area and/or that any trafficked parts have not been incorporated to public databases.

## Data Availability

Sequences have been uploaded to GenBank with the following accession numbers: PV872598–PV872614 and raw data have been deposited in the NCBI SRA under the following Bioproject: PRJNA1284350, and Biosamples: SAMN49719700–SAMN49719719. Data available from the Dryad Digital Repository [51]. Supplementary material is available online [52].
